# Bone health comparison in seven Asian countries using calcaneal ultrasound

**DOI:** 10.1186/1471-2474-14-81

**Published:** 2013-03-05

**Authors:** Marlena C Kruger, Joanne M Todd, Linda M Schollum, Barbara Kuhn-Sherlock, Drew W McLean, Kim Wylie

**Affiliations:** 1 Institute of Food, Nutrition and Human Health, Massey University, Private Bag 11222, Palmerston North 4442, Palmerston North, New Zealand; 2 Fonterra Co-operative Ltd, Private Bag 92032, Auckland, New Zealand; 3 Fonterra Research and Development Centre, Private Bag 11029, Palmerston North 4442, New Zealand

**Keywords:** Heel ultrasound, Asia, Risk for osteoporosis

## Abstract

**Background:**

Bone density measurements by DXA are not feasible for large population studies, whereas portable ultrasound heel scanners can provide a practical way of assessing bone health status. The purpose of this study was to assess bone health in seven Asian countries using heel ultrasound.

**Methods:**

Stiffness index (SI) was measured and T-scores generated against an Asian database were recorded for 598,757 women and 173,326 men aged over 21 years old using Lunar Achilles (GE Healthcare) heel scanners. The scanners were made available in public centres in Singapore, Vietnam, Malaysia, Taiwan, Thailand, Indonesia and the Philippines.

**Results:**

The mean SI was higher for men than women. In women SI as well as T-scores declined slowly until approximately 45 years of age, then declined rapidly to reach a mean T-score of < −2.5 at about 71–75 years of age. For men, SI as well as the T-score showed a slow steady decline to reach a mean of −2.0 to −2.5 at about 81–85 years. The results for females indicate that there are differences in the rate of decline between countries (significant differences between the slopes at P < 0.05). Vietnam had the fastest decrease for both T-Score and SI, resulting in this population having the poorest bone health of all countries at older ages. The results for males aged 46–85 years indicate that there are no significant differences in the rate of decline between countries for SI and T-Score. In both men and women aged 46–85 years, Vietnam and Indonesia have the lowest SI as well as T-Score for all age groups. For Vietnam and Indonesia, more than 50% of the women could be at risk of having osteoporosis and related fractures after the age of 70, while in Thailand and the Philippines this was >80 years.

**Conclusions:**

The heel scan data shows a high degree of poor bone health in both men and women in Asian countries, raising concern about the possible increase in fractures with ageing and the expected burden on the public health system.

## Background

According to the International Osteoporosis Foundation’s Asian Audit, published in 2009, the incidence of hip fracture has risen 2- to 3-fold in most Asian countries over the past 30 years [1-4]. Hip fracture rates in Hong Kong and Singapore are approaching those observed in US Caucasians [5]. The increased prevalence of osteoporosis- associated peripheral and vertebral fractures will also lead to an increase in socioeconomic burdens due to related costs for the public health system in the Asian region [2,5,6].

Bone mineral density using DXA is the standard diagnostic technique for osteoporosis but the cost is relatively high, and there is a shortage of DXA machines through most of the developing Asian countries [4]. Most of these machines are also located in the urban areas and therefore not accessible by the rural population. DXA is not ideal for community based studies, as DXA machines cannot be transported to rural areas and the cost of a scan is significant [7,8].

The general recommendation in Europe for DXA machines is 0.11 per 10,000 population (http://www.iofbonehealth.org)[4]. Most of the Asian countries are well below this ratio, with the ratio for Indonesia at 0.001 per 10,000 population for example, more than 70-fold lower [4]. As a result of this lack of available DXA technology, other clinical prediction tools have been developed, such as the Osteoporosis Self-assessment tool (OSTA) [9] and the Kohn Kaen Osteoporosis Study Score (KKOS) [10,11] in order to attempt to identify people at risk of osteoporosis and therefore who should receive a DXA scan. The FRAX® tool has been developed by the World Health Organisation (WHO) to evaluate fracture risk of patients [12]. It is based on individual patient models that integrate the risks associated with clinical risk factors as well as bone mineral density (BMD) at the femoral neck. But the FRAX® has not been validated for Asian populations with the exception of China, Hong Kong, the Philippines, Sri Lanka, Singapore and Taiwan [13].

Quantitative ultrasound (QUS) may offer an alternative tool for screening or assessment of risk of poor bone health in large populations. QUS measures the peripheral skeleton, and may give some assessment of bone microarchitecture in addition to bone mass [14]. It is relatively inexpensive, and is portable, and therefore could be used as a tool to screen for poor bone health at the community level [8]. QUS has also been shown to be as good as bone mineral density (BMD) assessed by DXA in predicting fracture risk [15]. Previous studies have shown that broad band attenuation (BUA) of QUS correlates moderately well with bone density by DXA [16]. The Lunar Achilles from GE Healthcare has been US FDA-approved for three clinical uses: It can predict the risk of hip fracture comparable to DXA at hip/spine; 2) it has valid T-scores for use in the same way as DXA at hip/spine, and 3) it has precision for monitoring bone changes in older populations (PMA number P970040).

There are very little data on the prevalence of low bone mass in several Asian countries. There have been population studies done in Taiwan including over 16,000 volunteers [14], Vietnam [8,17], and The Asian Osteoporosis Study (AOS) [1,5,18]. Lin et al. [14], reported mild to severe osteoporosis in 54% of the volunteers aged 50 and older living in Taiwan, while Hien et al. [8] and Thuy et al. [17] reported the prevalence of osteoporosis as 39% of women over the age of 50 years living in Hanoi City.

To date, there has not been a comprehensive assessment of bone health across the Asian region. The aim of the present study was to assess calcaneal stiffness index and T-scores using QUS in seven Asian countries from a large self-selected population. We hypothesised that the pattern of bone loss will be different between countries as well as between males and females.

## Methods

In this descriptive study, the Lunar Achilles Insight or Express (GE Healthcare, Madison, WI, USA) heel scanners were made available in several public centres such as shopping centres, community gatherings and office buildings in Singapore, Vietnam, Malaysia, Taiwan, Thailand, Indonesia and the Philippines between 2006 and 2009. Men and women attending the locations were invited to have their heel scanned. Only age and gender were recorded and there were no inclusion/ exclusion criteria in place, although all individuals signed a registration form. The individuals who volunteered to be scanned would have had to be ambulatory as they were visiting a public place. The information was gathered during public events and each individual consented to their information being stored.

Stiffness index (SI) was generated using broadband ultrasound attenuation and speed of sound measurements for 598,757 women and 173,326 men aged ≥ 20 years old. T-scores were then generated against the Asian reference population database provided with the heel scanners. The Achilles systems are non-invasive dry systems, which take 2–3 minutes to scan. For all subjects, the scan measured the right calcaneus.

Calibration was performed on each scan day according to manufacturer’s instructions. The Achilles systems use high frequency sound waves to evaluate bone status in the heel. They measure speed of sound (SOS) and broadband ultrasound attenuation (BUA) and combine them to form a clinical measure called the Stiffness Index. The manufacturers cited precision error for the SI measurement is 2.4%. As multiple scanners were used in various countries at the same time, they were not cross calibrated.

The SI was used to calculate a T-score based on a healthy young adult reference population. There have been 6 clinical studies involving 10,000+ women from which the reference population was created, and the ability to express and interpret results as T-scores were derived. This can be used to determine an individual’s risk of poor bone health. Therefore a T-score of > −1 was classified as normal, a score of < −0.1 and > −2.5 was classified as being at risk of having osteopenia while a T-score of < −2.5 was classified as at risk of having osteoporosis as per the FDA approval. The latter approach was reinforced by the position statement from the International Society for Clinical Densitometry (ISCD) [19]. While the QUS should not be used to diagnose osteoporosis, thresholds were defined as above to identify patients at high or low risk of having osteoporosis and being at risk of fracture.

Multiple studies using the Lunar Achilles QUS systems [19-22] have confirmed that the system has a 90% sensitivity for detecting osteoporosis at the spine and hip, using an Achilles T-Score referral threshold of −0.8 to −1.2. Using an Achilles T-score of −2.5 has been shown to provide a specificity of more than 90% for identifying only those subjects at high risk. Individuals with an Achilles T-score above −1.0 are considered at low risk for having osteoporosis, while those with an intermediate score of −1.0 to −2.5 may have some risk of low bone mass, and those with a T-score of < −2.5 are considered at high risk of having osteoporosis and a high fracture risk. These cut-offs were used to interpret the data obtained from the Achilles units used in this project.

### Data analysis

SI and T-scores were averaged for each 5 years age group and each country and are presented as mean and standard deviation for each age group and country as well as separately for males and females. Two-way Analysis of Variance (ANOVA) was used on the means to test for the effects of age and gender and their interaction on T-score and SI. Analysis of Covariance (ANCOVA) was used to compare the rates of decline in T-score and SI over age during the period of linear decline (age 46–85 years). Piecewise polynomial regression was used to test whether the rates of decline are similar in the 21 to 45 and 46–85 year age groups.

## Results

**Figure 1:**
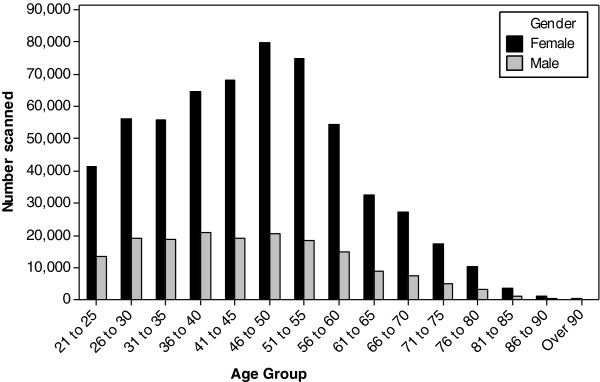
Number of males and females scanned per age group.

Figure 1 summarises the age and gender distribution of the participants. In total we report data from 598,757 heel scans for women, and 173,326 scans for men over the age of 20 years. The number of subjects per country and gender are presented in Table 1.

**Table 1:** Number of subjects per country and gender

**Country**	**Female**	**Male**	**All**
Indonesia	85864	36594	122458
Malaysia	120085	59458	179543
Philippines	105467	25527	130994
Singapore	16174	6563	22737
Taiwan	18309	6714	25023
Thailand	13280	3150	16430
Vietnam	239578	35320	274898
All	**598757**	**173326**	772083

**Figure 2:**
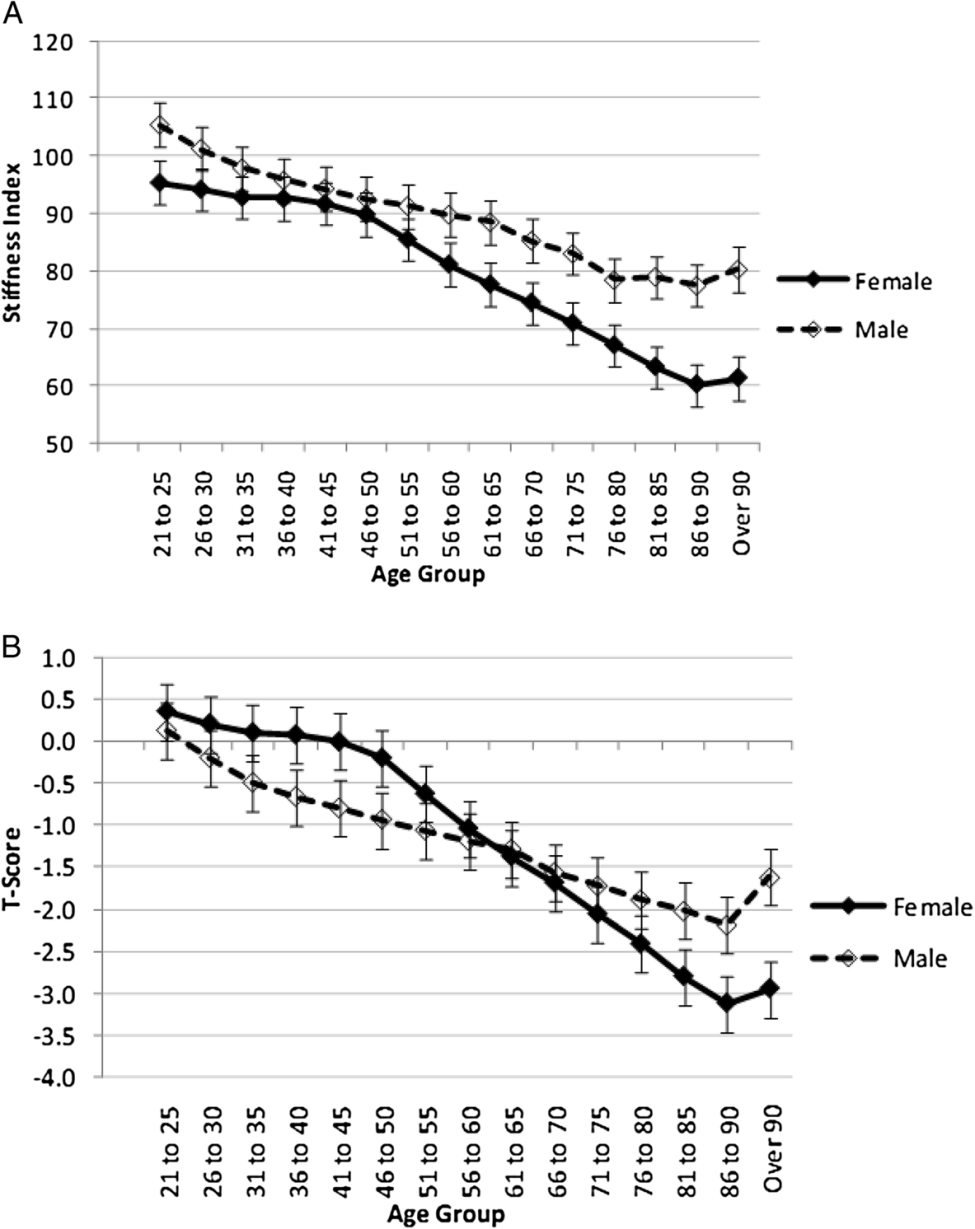
**A and B: Comparison of SI and T-scores between males and females.** Data is presented as LS mean and 95% confidence intervals.

Tables 2 and 3 and Figures 2A and B compare the rate of bone loss with age between men and women. The statistical analyses indicate that there is an interaction between age group and gender, indicating that the differences between men and women vary between age groups (Tables 2 and 3). The rate of loss is faster in the younger men compared to women aged between 21 and 55. Bone loss in the men is slow yet steady over time, while the rate of loss remains slow in the women, up until the age of 50 after which it increases rapidly. Women at age 55–60 reach a mean T-score of −1.0 which could indicate that many of these women may have low bone mass and could be at risk of having osteopenia [22]. The men already reach a mean T-score of −1.0 at age 46–50 which may indicate low bone mass at a much younger age compared to women.

**Table 2:** ANOVA for the effects of age and gender on stiffness index

**Source**	**DF**	**Type III SS**	**Mean Square**	**F Value**	**Pr > F**
Sex	1	4687.067	4687.067	186.7	<.0001
Age	14	22248.93	1589.209	63.3	<.0001
Sex*Age	14	1286.161	91.86863	3.66	<.0001

**Table 3:** ANOVA for the effects of age and gender on T-Score

**Source**	**DF**	**Type III SS**	**Mean Square**	**F Value**	**Pr > F**
Sex	1	0.001243	0.001243	0.01	0.9375
Age	14	178.8685	12.77632	63.37	<.0001
Sex*Age	14	21.42922	1.530658	7.59	<.0001

### Data for women aged 21-90+ years old

**Figure 3:**
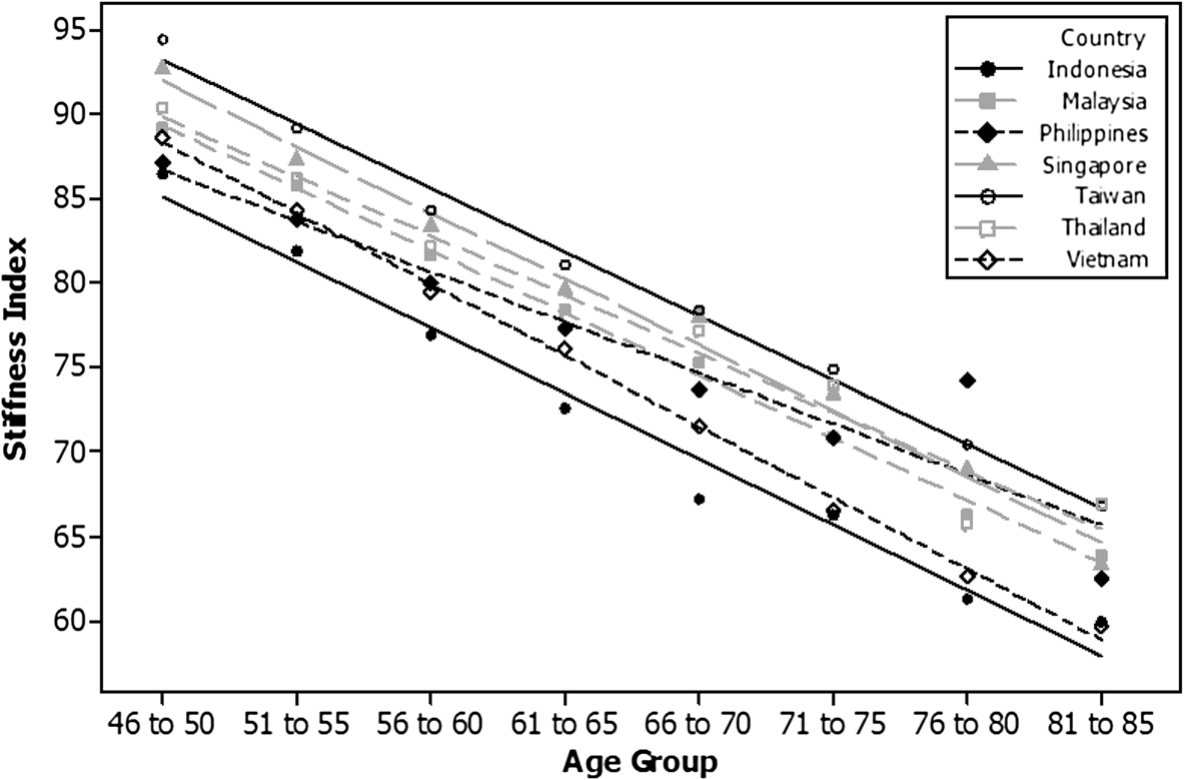
Rate of decrease in SI of women aged between 46 and 85 years.

Figure 3 shows the rate of decline in SI from the women scanned aged 46 to 85. The women from Indonesia in general have the lowest SI of all countries. The results for the women indicate that there is a significant difference in the rate of decline of SI between countries (P < 0.05) with Vietnam having a faster decline than the Philippines (0.84 units decline per additional year of age for Vietnam versus 0.6 units decrease per additional year of age for the Philippines). There is no difference in the rate of decline of the measured SI between the other countries. In the 46–50 year old age group, women from Indonesia have a significantly lower SI than Malaysia, Singapore, Taiwan and Thailand. In the same age group women from Taiwan have the highest SI, significantly higher than the mean SI for Indonesia, Philippines and Vietnam. In the 76 to 80 year age group, Vietnam and Indonesia have a significantly lower SI in comparison to all the other countries (P < 0.05); in the same age group, women from Taiwan has the highest SI but it is only significantly higher than those from Vietnam and Indonesia. There are no significant differences in the SI’s measured for this age group between Malaysia, Philippines, Singapore, Taiwan and Thailand.

The relevant T-scores are summarized in Table 4. The mean T-score for the young women at 20 years was low at between 0 and 0.5, and showed only a slight decline to approximately the age of 45, and then a rapid decline reaching a mean T-score of ≤ −2.5 at about 71–75 years of age. Women from Indonesia aged over 46 years, had significantly lower T-scores compared to all the other countries, but the rate of decline was faster in women aged 46–85 years from Vietnam. Women from Taiwan had the highest T-scores, significantly higher than Vietnam, Indonesia, Malaysia and Philippines but not Singapore and Thailand (P < 0.05).

**Table 4:** T-Score by country and age group – females (mean and standard deviation)

**Age Group**	**Indonesia**		**Malaysia**		**Philippines**		**Singapore**		**Taiwan**		**Thailand**		**Vietnam**	
21 to 25	−0.21	1.487	0.28	1.818	−0.17	1.703	0.86	2.085	0.76	2.089	0.46	1.812	0.45	2.102
26 to 30	−0.34	1.438	0.14	1.801	−0.38	1.720	0.66	1.960	0.77	2.008	0.33	1.760	0.24	1.965
31 to 35	−0.36	1.444	0.02	1.791	−0.20	1.751	0.30	1.910	0.61	1.804	0.19	1.699	0.14	1.982
36 to 40	−0.35	1.485	−0.07	1.726	−0.19	1.652	0.39	1.896	0.50	1.918	0.21	1.758	0.03	1.922
41 to 45	−0.43	1.488	−0.13	1.696	−0.22	1.699	0.26	1.884	0.56	1.890	−0.00	1.721	−0.12	1.927
46 to 50	−0.61	1.524	−0.28	1.675	−0.47	1.573	0.09	1.821	0.27	1.833	−0.12	1.651	−0.29	1.884
51 to 55	−1.05	1.475	−0.60	1.618	−0.79	1.649	−0.44	1.676	−0.26	1.709	−0.54	1.573	−0.73	1.785
56 to 60	−1.53	1.420	−1.01	1.568	−1.06	1.419	−0.83	1.599	−0.73	1.568	−0.95	1.567	−1.20	1.644
61 to 65	−1.92	1.468	−1.32	1.533	−1.27	1.685	−1.20	1.435	−1.06	1.508	−1.32	1.459	−1.55	1.627
66 to 70	−2.38	1.466	−1.62	1.571	−1.70	1.697	−1.37	1.631	−1.32	1.552	−1.45	1.549	−1.99	1.628
71 to 75	−2.52	1.473	−2.07	1.513	−2.06	1.490	−1.82	1.605	−1.68	1.593	−1.77	1.681	−2.51	1.518
76 to 80	−2.92	1.490	−2.50	1.516	−1.63	2.040	−2.27	1.787	−2.11	1.622	−2.59	1.403	−2.87	1.494
81 to 85	−3.04	1.522	−2.75	1.641	−2.89	1.380	−2.82	1.572	−2.43	1.576	−2.48	1.949	−3.18	1.495
86 to 90	−3.00	1.780	−2.83	1.564	−3.45	1.517	−3.57	1.421	−2.14	2.063	−3.26	1.266	−3.57	1.526
Over 90	−2.22	2.027	−1.93	2.754	−2.92	2.160	−2.70	^*^	−3.30	1.505	−3.90	^*^	−3.63	1.965

^*^indicates insufficient data.

### Data for men aged 20-90+ years old

**Figure 4:**
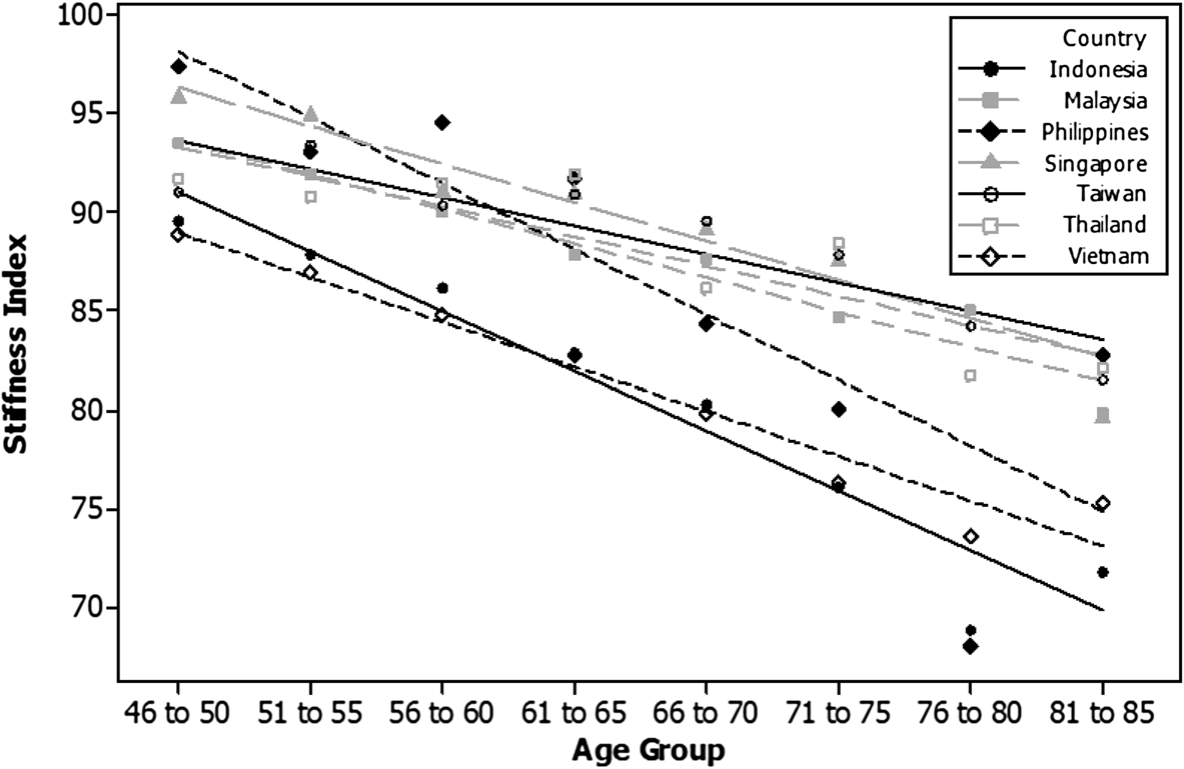
Rate of decrease in SI for men aged between 46 and 85 years.

Figure 4 shows the rate of decline in the SI in men aged between 46 and 85 years old. The rate of decline is the fastest in Indonesia and the Philippines: 0.60 and 0.67 units decrease per additional year of age versus 0.29 to 0.45 units decrease per additional year of age for the remaining countries. The difference in decline between Philippines, Taiwan and Thailand is marginally significant, but there is no difference in the decrease observed between any of the other countries. In the 46 to 50 year age group, Vietnam has the lowest SI and the Philippines the highest; this difference is significant (P < 0.05). All other countries are similar and none of the other differences are significant. In the 76 to 80 year age group, Indonesia has the lowest SI. In the same age group, Malaysia, Singapore, Taiwan, and Thailand have the highest SI, significantly higher than Indonesia. The Philippines and Vietnam have SIs below these countries but the difference is not statistically different.

The T-scores for men slowly decline from age 26–30 reaching a mean of ≤ −2.5 for some countries at about 81–85 years (Table 5). There are no significant differences between the rates of decline of the T-score between countries. The men from Indonesia and Vietnam had the lowest T-scores and greatest risk of poor bone health compared to the other countries but the rate of decline in the T-score (0.032 units decrease per additional year) between age 46 and 85 years was similar in all countries.

**Table 5:** T-Score by country and age group - males (mean and standard deviation)

**Age Group**	**Indonesia**		**Malaysia**		**Philippines**		**Singapore**		**Taiwan**		**Thailand**		**Vietnam**	
21 to 25	−0.14	1.387	0.18	1.703	−0.22	1.602	0.34	1.565	0.21	1.932	0.42	1.790	0.04	1.725
26 to 30	−0.46	1.391	−0.20	1.622	−0.42	1.451	−0.04	1.643	0.06	1.692	−0.03	1.728	−0.34	1.827
31 to 35	−0.66	1.349	−0.50	1.533	−0.75	1.719	−0.44	1.519	−0.06	1.883	−0.38	1.652	−0.68	1.639
36 to 40	−0.75	1.338	−0.64	1.502	−0.71	1.587	−0.54	1.518	−0.65	1.611	−0.40	1.671	−0.96	1.599
41 to 45	−0.79	1.367	−0.75	1.476	−0.76	1.532	−0.54	1.532	−0.82	1.517	−0.75	1.735	−1.16	1.506
46 to 50	−1.05	1.308	−0.86	1.465	−0.54	1.445	−0.71	1.532	−1.08	1.792	−1.04	1.467	−1.24	1.490
51 to 55	−1.24	1.324	−1.00	1.415	−0.97	1.628	−0.79	1.445	−0.89	1.490	−1.12	1.387	−1.40	1.512
56 to 60	−1.42	1.333	−1.15	1.442	−0.83	1.339	−1.10	1.427	−1.14	1.451	−1.06	1.477	−1.58	1.489
61 to 65	−1.69	1.373	−1.32	1.417	−1.01	1.422	−1.11	1.405	−1.11	1.490	−1.02	1.444	−1.73	1.499
66 to 70	−1.96	1.398	−1.35	1.455	−1.64	1.872	−1.26	1.484	−1.22	1.387	−1.51	1.468	−1.99	1.497
71 to 75	−2.23	1.353	−1.59	1.488	−1.93	1.259	−1.40	1.381	−1.34	1.610	−1.32	1.392	−2.22	1.492
76 to 80	−2.53	1.374	−1.56	1.497	−1.65	1.954	−1.41	1.686	−1.67	1.367	−1.87	1.553	−2.49	1.562
81 to 85	−2.32	1.435	−1.99	1.664	−1.77	1.337	−2.06	1.194	−1.87	1.360	−1.84	1.520	−2.28	1.736
86 to 90	−2.78	1.211	−1.41	1.738	−3.16	1.019	−1.92	1.955	−0.67	1.971	−2.27	1.872	−3.08	1.416
Over 90	−2.48	1.642	−1.22	1.977	−1.53	2.003	0.17	0.666	−3.02	2.220	^*^	^*^	−3.27	1.182

^*^indicates insufficient data.

## Discussion

Several studies have now shown that QUS at peripheral sites can be used as a screening tool to assess bone health [8,14,17,20,21]. Speed-of-sound measurements at the calcaneus can identify persons at risk of osteoporotic fracture as reliably as bone mineral density measurements [22,23] and could be an ideal tool to screen for osteoporosis at the community level [24-26]. In the current study we aimed to assess bone health in several Asian countries using the GE Achilles Insight or Express machine, and then to comment on the prevalence of low bone mass in men and women aged between 20 and 90+ years in these countries. The SI and T-scores were generated by the units, and the ISCD criteria [19] were used to classify people at risk of poor bone health. Our data show a high degree of poor bone health in both men and women in the seven Asian countries where we conducted the assessments.

The younger males in general had a higher SI compared to females aged between 21–40 while the T-scores for both men and women aged between 20 and 25 were between 0 and 0.5. Bone loss was slow in the women up to the age of 45–50, after which it increased significantly, with more than 50% of the women being at risk of being osteoporotic at age 70+ years. In men, bone loss was at a similar rate from age 20 to 90 years with more than 50% being classified as being at risk of being osteopenic or osteoporotic at age 80+ years. Similar differences in rates of bone loss were also reported by Lin et al. [14] in a Taiwanese population which also had an increased rate of bone loss in women compared to men, after the age of 60 years.

The prevalence of low bone mass was highest in Indonesia for both women and men. In the 46–50 age groups, Indonesia had a significantly lower SI than Malaysia, Singapore, Taiwan and Thailand. In Indonesia up to 70% of women and men over 50 years could be classified as at risk of being either osteopenic or osteoporotic. Data from the Indonesian Osteoporosis society (PEROSI) suggest that about 41.8% of men and 90% of women are osteopenic, while 28.8% of men and 32.3% of women have osteoporosis as per the WHO criteria [4,19]. These data do not define the age groups and can therefore not be directly compared to our study. Our published data on a small cohort of postmenopausal women in Indonesia revealed that 66.3% could be classified as being either osteopenic or osteoporotic, according to DXA of the lumbar spine [27].

In Taiwan the percentage of women over 50 years with low bone mass was 47.5% while the percentage for men was 57.1%. Chie et al. [28] reported that the incidence of hip fracture in Taiwanese women over 50 years was similar to those recorded for Western countries, but that the age-specific incidence of hip fracture of elderly Taiwanese men was higher than in US Caucasian men, at about 65% that for women. The QUS data for the Philippines indicate a prevalence of low bone mass in 59.3% in women over 50 years and 56.6% in older men. In a previous study using DEXA, we found 67.2% of postmenopausal women had low bone mass in the lumbar spine [27]. However, this was a small study including only 58 women, and so may not be representative of the total population. Miura et al. [29] reported a 19.8% prevalence of osteoporosis in urban postmenopausal women in the Philippines, using QUS. Our data using QUS indicate the percentage at risk of osteoporosis to be 22.1%, similar to the data reported by Miura et al. [30].

Our data from Thailand could have underestimated the prevalence of low bone mass, as differences in the age ranges in various publications make direct comparisons difficult. Pongchaiyakul et al. [31], reported the prevalence of osteoporosis by femoral neck or lumbar spine BMD using DXA, to be 33% in women older than 60 years, while we report that up to 17% of women over 60 years could be at risk of having osteoporosis and another study showed the prevalence of osteoporosis to be 50% in women over 70 years old [32]. As mixed results have been reported for Thai women, Pongchaiyakul et al. [33] examined the prognostic value of combining QUS with clinical risk factors, using a cohort of women aged between 38 and 85 years, and found the prevalence of osteoporosis to be 12.7% in this group of women. Age, weight and QUS outcomes were significantly associated with osteoporosis risk. The latter study suggests that a combination of QUS with age and weight could be used to create a normogram to be used to estimate the risk for poor bone health in Asian women.

The reported data raise a concern for bone health in the wider Asian region. Hip fracture rates in Hong Kong and Singapore have been reported to be approaching those recorded for Caucasians [5], and while the rates are lower for countries such as Malaysia and Thailand, they are likely to increase. In China, at this time, more than 69 million people over the age of 50 suffer from osteoporosis, with 687,000 hip fractures each year. WHO estimated that more than 50% of hip fractures will occur in Asia by 2050 [6], and the number of people with hip fracture in Asia will be about 3.2 million per year [2,6]. A recent review by Cooper et al. [34] suggested that while hip fracture rates may be reaching a plateau in the Western world, there is an increasing age-adjusted incidence rate of hip fractures among Asian men and women. The cost associated with hip fractures is substantial; the combined annual cost of all osteoporotic fractures has been estimated to be $20 billion in the USA, and 30 million Euros in the European Union [34].

QUS can predict the risk of wrist and osteoporosis-related fractures [35], the risk of vertebral fractures [23,25,36], the risk of hip fractures [15,37,38] and can discriminate between women with and without vertebral fractures [23,38] and hip fractures [22,35,37]. Heel QUS is also strongly correlated with the strength of the proximal femur [39]. Our data could therefore indicate that the risk of osteoporotic fracture is high in women and men over the age of 70 years living in the seven countries where we collected data.

Risk factors for low bone mass in Asia include low calcium in the diet, and relatively high occurrence of vitamin D insufficiency. Mean daily calcium intakes in the seven countries vary between just above 200 mg in Indonesia and Thailand [27,40] to about 450 mg for Malaysia, Singapore and Taiwan [18,41]. Suboptimal vitamin D status has also been widely reported for many Asian countries. We reported mean 25 (OH) vitamin D_3_ levels for postmenopausal women in Jakarta, Indonesia to be 45.06 nmol/L (range 41.02 - 49.09) and for women living in Manila, Philippines to be 62 nmol/L (range 56.2 - 67.7) [27]. Other reported values are 52 nmol/ L for Thailand [4], and 44 nmol/L for postmenopausal women living in Malaysia [42]. In all of the above mentioned studies, a significant negative correlation was reported between serum 25 (OH) vitamin D_3_ and Parathyroid hormone (PTH) levels [40,42,43]. There is also a strong relationship between suboptimal vitamin D status, high PTH levels and the risk for hip fractures [43-46].

The present study had limitations: firstly, none of the QUS bone density measurements were validated against a DXA measurement for the same person; secondly, the QUS data can only identify people at risk of low bone mass, and is not a diagnostic tool. Thirdly, participants were self-selected and primarily from urban areas. And lastly, measuring bone density using QUS does not capture the Z-score which may have been more informative for the younger populations; the SI as well as generated T-scores were used as proxy measurements.

## Conclusions

The results presented here provide a snapshot of the bone health status of participants from seven regions in South East Asia. Overall results indicate that there is extensive poor bone health in both males and females living these regions. The measured SI’s indicate low bone mass even among young men and women, and the patterns of bone loss were very similar. We conclude that the data presented may indicate that the risk of having low bone mass and osteoporotic fractures is high in women and men over the age of 70 years living in these countries with the highest risk being for people living in Indonesia and Vietnam.

There is a strong need to continue to develop detailed, robust evidence of bone health status in communities throughout South East Asia. Osteoporosis has a severe effect on the quality of life and independence of sufferers, and is a considerable socio-economic burden for individuals, communities and the public health systems [4,5]. Studies such as these will contribute to an accurate assessment of bone health and its prevalence which will help to provide valuable information for the development and implementation of tailored health promotion campaigns, improved patient care, and reduced economic burdens.

## Competing interests

MC Kruger, K Wylie - none.

JM Todd, LM Schollum, B Kuhn-Sherlock, DW McLean - employed by Fonterra Co-operative Group Ltd.

## Authors’ contributions

MK was the primary author; JT and LMS edited the manuscript, BKS was responsible for the statistical analyses and graphics; DWM and KW were responsible for data capture and entry. All authors read and approved the final manuscript.

## Pre-publication history

The pre-publication history for this paper can be accessed here:

http://www.biomedcentral.com/1471-2474/14/81/prepub
